# Supplementary material for: Iterative improvement in the automatic modular design of robot swarms
Source: PeerJ Comput Sci. 2020 Dec 7;6:e322. doi: 10.7717/peerj-cs.322 (PMC7924708; doi:10.7717/peerj-cs.322)
Supplement: Supplemental Information 3 [file peerj-cs-06-322-s003.zip › argos3/doc/api/standalone/a00456.html]

ARGoS: plugins/robots/foot-bot/simulator/footbot\_base\_ground\_rotzonly\_sensor.cpp File Reference


- Main Page
- Related Pages
- Namespaces
- Classes
- Files

- File List
- File Members

# plugins/robots/foot-bot/simulator/footbot\_base\_ground\_rotzonly\_sensor.cpp File Reference

`#include <argos3/core/simulator/simulator.h>`  
`#include <argos3/core/simulator/entity/composable_entity.h>`  
`#include <argos3/core/simulator/entity/embodied_entity.h>`  
`#include <argos3/core/simulator/entity/floor_entity.h>`  
`#include <argos3/plugins/simulator/entities/ground_sensor_equipped_entity.h>`  
`#include "footbot_base_ground_rotzonly_sensor.h"`  

Include dependency graph for footbot\_base\_ground\_rotzonly\_sensor.cpp:

Go to the source code of this file.

|  |  |
| --- | --- |
| Namespaces | |
| namespace | argos |

|  |  |
| --- | --- |
|  | The namespace containing all the ARGoS related code. |

| Functions | |
|  | argos::REGISTER\_SENSOR (CFootBotBaseGroundRotZOnlySensor,"footbot\_base\_ground","rot\_z\_only","Carlo Pinciroli [ilpincy@gmail.com]","1.0","The foot-bot base ground sensor.","This sensor accesses the foot-bot base ground sensor. For a complete description\n""of its usage, refer to the ci\_footbot\_base\_ground\_sensor.h interface. For the XML\n""configuration, refer to the default ground sensor.\n","Usable") |

---

Generated on 10 Jul 2018 for ARGoS by 
 1.6.1
